# Characterization of the GBoV1 Capsid and Its Antibody Interactions

**DOI:** 10.3390/v13020330

**Published:** 2021-02-20

**Authors:** Jennifer Chun Yu, Mario Mietzsch, Amriti Singh, Alberto Jimenez Ybargollin, Shweta Kailasan, Paul Chipman, Nilakshee Bhattacharya, Julia Fakhiri, Dirk Grimm, Amit Kapoor, Indrė Kučinskaitė-Kodzė, Aurelija Žvirblienė, Maria Söderlund-Venermo, Robert McKenna, Mavis Agbandje-McKenna

**Affiliations:** 1 Department of Biochemistry and Molecular Biology, Center for Structural Biology, College of Medicine, University of Florida, Gainesville, FL 32610, USA; jennifer.yu@ufl.edu (J.C.Y.); mario.mietzsch@ufl.edu (M.M.); amritisingh@ufl.edu (A.S.); albertojimenezy@cop.ufl.edu (A.J.Y.); shwetakailasan@gmail.com (S.K.); pchipman@ufl.edu (P.C.); rmckenna@ufl.edu (R.M.); 2 Biological Science Imaging Resource, Department of Biological Sciences, Florida State University, Tallahassee, FL 32306, USA; nilakshee.bhattacharya@duke.edu; 3 Department of Infectious Diseases/Virology, Medical Faculty, BioQuant, University of Heidelberg, 69120 Heidelberg, Germany; julia.fakhiri@hotmail.com (J.F.); dirk.grimm@bioquant.uni-heidelberg.de (D.G.); 4 Center for Vaccines and Immunity, The Research Institute at Nationwide Children’s Hospital, Columbus, OH 43220, USA; Amit.Kapoor@Nationwidechildrens.org; 5 Department of Immunology and Cell Biology of the Institute of Biotechnology of Vilnius University, 10257 Vilnius, Lithuania; indkuc@ibt.lt (I.K.-K.); azvirb@ibt.lt (A.Ž.); 6 Department of Virology, University of Helsinki, 00014 Helsinki, Finland; maria.soderlund-venermo@helsinki.fi

**Keywords:** bocavirus, capsid, parvovirus, cryo-EM, gene therapy, antigenicity

## Abstract

Human bocavirus 1 (HBoV1) has gained attention as a gene delivery vector with its ability to infect polarized human airway epithelia and 5.5 kb genome packaging capacity. Gorilla bocavirus 1 (GBoV1) VP3 shares 86% amino acid sequence identity with HBoV1 but has better transduction efficiency in several human cell types. Here, we report the capsid structure of GBoV1 determined to 2.76 Å resolution using cryo-electron microscopy (cryo-EM) and its interaction with mouse monoclonal antibodies (mAbs) and human sera. GBoV1 shares capsid surface morphologies with other parvoviruses, with a channel at the 5-fold symmetry axis, protrusions surrounding the 3-fold axis and a depression at the 2-fold axis. A 2/5-fold wall separates the 2-fold and 5-fold axes. Compared to HBoV1, differences are localized to the 3-fold protrusions. Consistently, native dot immunoblots and cryo-EM showed cross-reactivity and binding, respectively, by a 5-fold targeted HBoV1 mAb, 15C6. Surprisingly, recognition was observed for one out of three 3-fold targeted mAbs, 12C1, indicating some structural similarity at this region. In addition, GBoV1, tested against 40 human sera, showed the similar rates of seropositivity as HBoV1. Immunogenic reactivity against parvoviral vectors is a significant barrier to efficient gene delivery. This study is a step towards optimizing bocaparvovirus vectors with antibody escape properties.

## 1. Introduction

Gorilla bocavirus 1 (GBoV1) is a member of the genus *Bocaparvovirus* in the *Parvoviridae* that contain single-stranded DNA (ssDNA) packaging viruses [1]. The family is divided into three subfamilies, including the subfamily *Parvovirinae* whose members infect vertebrate hosts [1]. Bocaparvoviruses represents the largest genus in this subfamily, with 21 classified species that infect a variety of hosts, including cows, rabbits, rodents, humans and non-human primates [2,3,4,5,6,7,8,9,10]. Bovine parvovirus (BPV) is the first discovered member of the genus, isolated from cattle in 1959 [3]. The first discovered member infecting humans is human bocavirus 1 (HBoV1), isolated in 2005 from nasopharyngeal aspirates of children under 2 years of age with acute respiratory infections [7]. Enteric strains, HBoV2-4, were then described from children with acute gastroenteritis [8,9]. GBoV1 was isolated from gorillas with enteritis and is the first identified non-human primate bocavirus [10].

The bocaviruses have a ~5.5 kb genome that consist of three open reading frames (ORFs), the *ns* gene on the left end, the *cap* gene on the right end and the *np* gene between *ns* and *cap* [7]. The three ORFs are flanked by two non-identical hairpin structures and are transcribed from a single promoter (p5) to generate a single pre-mRNA that is alternatively spliced [11,12]. The *ns* gene encodes non-structural proteins that are essential for viral DNA replication [11]. The *np* gene encodes the NP1 protein, which was shown to be play a role in pre-mRNA processing and subsequent capsid protein expression [11,13]. The structural proteins that form the viral capsid, VP1, VP2 and VP3, are encoded by the *cap* gene [14]. Sixty copies of VP1, VP2 and VP3 assemble one T = 1 icosahedral capsid in an approximate 1:1:10 ratio in 2-, 3-, 5-related symmetries [14,15]. The three VPs share the same C-terminus. VP3 is the major capsid protein and is the smallest of the three VPs. VP2 shares a common region with VP1 at the N-terminus, called the VP1/2 common region [16]. The unique region of the minor VP1 protein N-terminus (VP1u) contains a phospholipase activity (PLA2) shown to be responsible for endosomal escape during trafficking to the nucleus and is absolutely required for infectivity [17,18,19]. The VP1u is hypothesized to externalize through a channel located at the 5-fold axis of the capsid to activate its PLA2 activity [20,21]. While VP1 incorporation is essential for viral infectivity, the VP3 protein alone was shown to form intact capsids, termed VP3-only capsids, with similar antigenicity to wild-type capsids [22,23]. It is the capsid that interacts with the host environment and is a determinant of host and cell recognition, host immune response and cell entry [24].

Previously, the capsid structures of HBoV1-4 have been reported that showed conserved features across the *Parvoviridae* family, as well as features unique to the genus [23,25]. The bocavirus VP monomer has a conserved eight-stranded β-barrel motif (βB to βI), forming the interior of the capsid, with a βA strand that runs antiparallel to βB and an α-helix (αA) located between strands βC and βD of the β-barrel [16]. The loops between the β strands form the surface of the capsid and these surface loops are labeled based on the flanking β strands, for example, the loop between βD and βE is the DE loop. Within the loops are defined variable regions (VRs), ranging from VR-I to VR-IX. These variable regions (VRs) were previously defined for the bocaviruses based on comparisons to the structure of bovine parvovirus (BPV), the first capsid structure solved from the *Bocaparvoviridae* genus [26]. In addition to the conserved β-barrel motif, βA and αA, the bocaviruses were reported to possess a unique α-helix (αB) located near VR-III as well as a basket-like structure beneath the 5-fold channel [23].

Recently, HBoV1 and GBoV1, along with enteric human strains HBoV2-4, were proposed as gene therapy delivery vectors. The interest in HBoV1 stems from its specific tropism for the apical side of polarized human airway epithelia (pHAE), which is optimal for the treatment of cystic fibrosis [27,28]. Gene therapy has been the “gold standard” for the treatment of monogenetic diseases such as cystic fibrosis but Adeno-associated virus serotype 2 (AAV2), a vector used for treatment was shown to be inefficient at delivery to the lung and has tropism for the basolateral side of pHAE [29,30,31]. In addition to the favorable tropism of HBoV1, the expanded genome capacity of bocaviruses (5.5 kb), compared to AAV, allows the packaging of the full-length 4.7 kb *CFTR* gene [7,28]. Due to these advantages, various studies have aimed to optimize a recombinant (r)AAV2/HBoV1 pseudotyped vector, a HBoV1 capsid-based vector that packages a transgene with rAAV2 inverted terminal repeats (ITRs), for delivery of the *CFTR* gene. This vector was shown to have tropisms similar to the HBoV1 wild-type virus [28,32,33].

Despite the advantages of HBoV1 as a gene therapy vector, the capsid has high seroprevalence in the human population, a hurdle to therapeutic gene delivery. GBoV1 is an alternative to HBoV1, as it is capable of also infecting the apical side of pHAE efficiently, package 5.5 kb and less susceptible to neutralization by pooled intravenous immunoglobulin (IVIgs) [33]. The goal of this study was to characterize the GBoV1 capsid and to better understand the functional regions including antigenicity of the capsid. We report the high-resolution structure of the GBoV1 capsid, determined by cryo-electron microscopy (cryo-EM) and 3D single-particle reconstruction. The GBoV1 VP3 monomer conserves features common to parvoviruses and contains features unique to bocaviruses, for example, α-helix αB. Within the capsid interior is a 5-fold basket-like density, which appears smaller when compared to HBoV1-4. The main differences between the HBoV1 and GBoV1 monomer are localized to VR-I, VR-III and VR-V. Low-resolution structures of the GBoV1 capsid complexed with mouse monoclonal antibodies (mAbs) 15C6 and 12C1, originally generated against HBoV1, show epitopes localized to the 5-fold and 3-fold axes, respectively, in agreement with reports for HBoV1 [22]. However, two other mAbs, targeted at the HBoV1 3-fold axis, did not recognize GBoV1 indicating structural variation at this capsid region between the two viruses. Both viruses showed comparable high seroprevalence against human sera suggesting a high degree of cross-reactivity for the sera tested and a conserved capsid region, such as the 5-fold region, as forming the epitopes. These observations begin to unravel the antigenic properties of GBoV1 and provide information that could aid engineering vectors with reduced antigenic reactivity and, thus, therapeutic efficacy.

## 2. Methods

### 2.1. Virus-Like Particle Production and Purification

The Bac-to-Bac baculovirus system was used for the expression of HBoV1, HBoV4, GBoV1, AAV2 and AAV5 virus-like particles (VLPs) as described previously for HBoV1 (VP3 only), AAV2 and AAV5 [22,34]. For the generation of GBoV1 expressing VP1, VP2 and VP3 (termed wild-type), the entire *cap* gene (NCBI accession no. NC_014358.1) was cloned and inserted into the pFastBac plasmid with an ACG start codon for VP1. For the generation of GBoV1 VP3-only VLPs, the VP3-encoding sequence (without the VP1 unique region and the VP1/2 common region) was directly cloned and inserted into the pFastbac plasmid for transposition into the baculovirus expression vector. Based on the standard manufacturer’s protocol, the baculovirus expression vectors were then used to generate recombinant baculovirus stocks expressing GBoV1 wild-type and VP3-only VLPs [35]. Briefly, *Sf*9 insect cells, maintained in SFM Sf9-900 medium (Thermo Fisher, Waltham, MA, USA) with 10% fetal bovine serum (FBS) and antibiotics, were infected at a multiplicity of infection (MOI) of 5 and harvested 72 h post-infection. Sucrose cushion and sucrose density gradients were performed for the purification of VLPs after three rounds of freeze-thaw cycles and benzonase (Millipore) treatment of the cell pellets, as described previously [15]. Purified samples were dialyzed into 1× phosphate-buffered saline (PBS) (2.8 mM KCl, 137 mM NaCl, 10 mM Na_2_HPO_4_, 1.8 mM KH_2_PO_4_) and concentrated to 0.5–2 mg/mL using Apollo concentrators (Orbital Biosciences, Topsfield, MA, USA). Purity and capsid integrity of the VLPs was confirmed by sodium dodecyl sulfate polyacrylamide gel electrophoresis (SDS-PAGE) and negative stain electron microscopy analysis using a Tecnai G2 Spirit electron microscope (FEI, Hillsboro, OR, USA) at 120 kV, respectively, as described previously [22].

### 2.2. Dot Immunoblot Analysis

In dot immunoblots HBoV1 VP3-specific mAbs were probed against GBoV1 (VP3 only and WT), HBoV1 (VP3 only), HBoV4 (VP3 only), AAV2 and AAV5 native capsids. To determine the seroprevalence, human sera from healthy donors (Valley Biomedical, Winchester, VA, USA) were utilized in dot immunoblots against GBoV1, HBoV1 (VP3 only), AAV2 and AAV5. H1-H1 polyclonal antibody (rabbit double-immunized with HBoV1 VP3 VLPs) was used against denatured capsids as a positive BoV control [36]. Dot blots were performed on nitrocellulose membrane dipped in 1× PBS. Denatured (incubating capsids at 100 °C for 5 min) and non-denatured VLPs were applied directly to the membrane at approximately 10 ng^−1^ μg using a vacuum manifold, letting the sample incubate for 10 min. The membranes were blocked in 6% milk in 1× PBS overnight at 4 °C. For the primary antibody, mAb 15C6 was added at a 1:2000 dilution; mAbs 12C1, 4C2 and 9G12 [22] were added at a 1:1000 dilution; H1-H1 was added at a 1:1000 dilution; human sera samples were added at a 1:500 dilution in 6% milk in PBS-T (PBS with 0.1% Tween) and incubated on the membrane for 1.5 h at room temperature (RT). Secondary anti-mouse and anti-rabbit antibodies were applied to the membrane in a 1:5000 dilution and anti-human IgG was applied in a 1:50,000 dilution in 6% milk in PBS-T for 1 h at RT. Five min washes were performed three times before and after incubation with secondary antibody. Blocking, primary and secondary antibody steps were all performed on a shaker. Finally, luminol substrate was applied to the membrane and incubated in the dark for 1 min, before the membrane was exposed to X-ray radiography film and developed. 

### 2.3. Generation of Fab Fragments

Production of the 15C6, 12C1, 4C2 and 9G12 mAbs from BALB/c mice injected with HBoV1 VLPs and subsequent purification of IgGs were previously described [22]. IgG from 15C6 and 12C1 at a concentration of approximately 1–2 mg/mL was buffer-exchanged into 20 mM sodium phosphate, pH 7.0, 10 mM EDTA for papain cleavage. Papain was added to the IgG samples and incubated for 16–20 h at 37 °C with rotation. The papain-IgG mixture was centrifuged at a low speed (1000× *g*) and the supernatant containing cleaved Fab fragments was loaded onto a protein A column. The Fc portion of the cleaved IgG was captured in the protein A column and flowthrough containing the desired Fab fragments was collected. This flowthrough was concentrated to ~0.5 mg/mL in an Apollo concentrator with a 9 kDa molecular mass cutoff. sodium dodecyl sulfate-polyacrylamide gel electrophoresis (SDS-PAGE) was performed to confirm the purity of the Fab fragment sample.

### 2.4. Preparation of GBoV1-Fab Complexes and GBoV1 VLPs for Cryo-EM Data Collection

GBoV1 VLPs were mixed with the 15C6 and 12C1 Fab fragments at a 1:120 to 1:180 (VLP:Fab) ratio to ensure binding-site saturation. The VLP: antibody complexes were incubated on ice for 30 min to 1 h and 3 μL of sample was vitrified onto C-flat holey carbon grids (Protochips, Inc.) using the Vitrobot Mark IV (FEI Co.). For the GBoV1 VLPs (wild-type and VP3-only), 3 μL of sample was prepared as described for the complexes. 

### 2.5. Cryo-EM Data Collection 

For the GBoV1-Fab complexes, micrographs were collected from frozen grids using a Tecnai G2 F20-TWIN transmission electron microscope (FEI) with a 200 kV voltage under low-dose conditions (20 e^−^/Å^2^) at a magnification of 82,500× on a 16-megapixel charge coupled device (CCD) camera with pixel size 15 μm, resulting in micrographs with a pixel size of 1.82 Å. This microscope and camera were also used for screening grids for ice quality and particle distribution of GBoV1 prior to high-resolution data collection and also for collecting a low-resolution data set for comparison to the complex structures. For the GBoV1 VLP-alone high-resolution studies, holey carbon grids with vitrified VLPs were used to collect micrograph movie frames on a Titan Krios electron microscope (FEI Co.) operated at 300 kV with a K3 DED using the Leginon application. High-resolution data collection was performed with a total dose of 60 to 67 e^−^/Å^2^ for up to 50 movie frames per micrograph. The movie frames collected on the K3 detector were aligned using MotionCor2 with dose weighting as previously described [37]. Data sets were collected as part of the NIH project “Southeastern Center for Microscopy of MacroMolecular Machines” (SECM4).

### 2.6. 3D Particle Reconstruction

The cisTEM software package was used for three-dimensional (3D) image reconstruction of both, wild-type and antibody-complex structures [38]. The aligned micrographs were first imported and their microscope-based contrast transfer function (CTF) estimated. Suboptimal-quality micrographs were eliminated. Capsids on the remaining micrographs were automatically selected using a particle radius of 125 Å. The selected capsids were subjected to 2D classification and undesirable classes, such as ice or impurities, were removed from the dataset. Both, *ab-initio* 3D reconstruction and automatic refinement was performed under default settings. *Ab-initio* 3D reconstruction generated an initial low-resolution model with 10% of the total boxed particles with imposed icosahedral symmetry and automatically refined with the entire dataset. Map sharpening of the high-resolution structure used a pre-cut off B-factor value of 90 Å^2^ and variable post-cut off B-factor values such as 0, 20 and 40 Å^2^. Using the UCSF-Chimera software, the sharpened density maps were analyzed and the −90 Å^2^/0 Å^2^ map was used for further model building and structure refinement. The final resolution of the structures was estimated based on a Fourier shell correlation (FSC) threshold criterion of 0.143 (Table 1).

### 2.7. Model Building and Structure Refinement

The 3D model for GBoV1 wild-type VP3 monomer was generated from the protein sequence (NCBI accession ADK34012.1) in the online program SWISS-MODEL using the structure of HBoV1 (Research Collaboratory for Structural Bioinformatics [RCSB] PDB code 5URF) as a template [39]. This reference monomer model was used to generate a 60mer (based on 60 copies of the VP3 protein) with the VIPERdb2 oligomer generator [40] and docked as rigid bodies into the GBoV1 density map using the “fit in map” subroutine in UCSF-Chimera [41]. The docked VP monomer model was adjusted to better fit the GBoV1 wild-type cryo-reconstructed density map with manual model-building tools and real-space-refine options in Coot [42]. Further refinement was performed on the model with PHENIX, using the real-space-refinement subroutine under default settings for five macrocycles [43]. This refined model was inspected in Coot and amino acid side chains are adjusted, if needed, for favorable statistics. After another round of refinement in PHENIX, an icosahedral model was generated from 60 copies of the refined VP3 monomer with the VIPERdb2 oligomer generator. The 60-mer VP3 monomer was further refined in PHENIX using B-factor refinement options.

### 2.8. Antibody Epitope Mapping

The high-resolution 60-mer GBoV1 structure was rigid body-docked into the antibody-complex density maps, using the “fit in map” subroutine in UCSF-Chimera. A generic Fab (PDB ID: 2FBJ) was fitted into the density of the Fab using the same subroutine in UCSF-Chimera [41]. The resulting pseudo-atomic model was used to generate a roadmap using RIVEM [44]. The contact residues, residues in the interface between the capsid and antibody structure, were identified via manual inspection in the program Coot [42]. Occluded residues were also identified manually by generating a roadmap in RIVEM using the GBoV1 structure and generic Fab. 

### 2.9. Sequence and Structural Comparison

The VP3 models of HBoV1-4 and GBoV1 were analyzed in Coot and using the superposition tool. Overall paired root mean squared deviations (RMSD) were calculated between Cα positions. The distances between Cα positions of regions with insertions or deletions were manually measured in Coot with the distance tool. Regions with two or more adjacent amino acids and a greater than 2 Å difference determined by Coot are considered to be structurally diverse and are assigned to previously described VRs [42]. 

### 2.10. Structure Accession Numbers 

The GBoV1 WT cryo-EM reconstructed density map and model built for the capsid were deposited in the Electron Microscopy Data Bank (EMDB) with accession numbers EMD-23460 and PDB ID 7LNK, respectively.

## 3. Results & Discussion

### 3.1. GBoV1 Shares Conserved Capsid Features with the HBoVs

**Figure 1:**
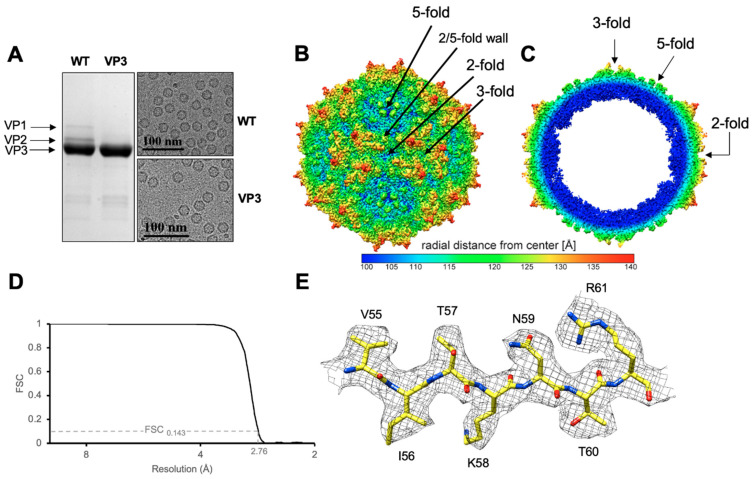
The capsid structure of Gorilla bocavirus 1 (GBoV1). (**A**) sodium dodecyl sulfate-polyacrylamide gel electrophoresis (SDS-PAGE) of GBoV1 WT and VP3 only samples confirming the presence of VP1, VP2 and VP3 (~80, 65, 60 kDa) and cryo-electron micrograph showing intact viral particles. (**B**) Capsid density map of GBoV1 WT contoured at sigma (σ) threshold of 1.0. The radial distance from the center measured in Å is colored as shown. Arrows point to the 5-fold, 3-fold or 2-fold symmetry axis and the 2/5-fold wall. (**C**) Cross-sectional view of GBoV1 WT density map. (**D**) Fourier shell correlation (FSC) plot for the cryo-reconstruction with an estimated resolution of 2.76 Å at an FSC threshold of 0.143. Resolution (Å) is presented using a log scale. (**E**) Atomic model of amino acids 55–61 (βB) represented within their density map contoured at a σ threshold level of 1. C = yellow, O = red and N = blue. Panels B, C and E were made using UCSF-Chimera [41].

The GBoV1 VLPs were produced using recombinant baculovirus expressing either GBoV1 VP1, VP2 and VP3 (GBoV1 WT) or only VP3 (GBoV1 VP3-only) in *Sf*9 insect cells. The purified GBoV1 WT sample was analyzed on SDS-PAGE to confirm the presence and purity of VP1, VP2 and VP3 with a corresponding molecular weight of approximately 60, 65 and 80 kDa. For GBoV1 VP3-only, only one band is present at 60 kDa (Figure 1a). Cryo-EM micrographs confirmed the presence of intact capsids with a diameter of approximately 250 Å without the presence of contaminants (Figure 1a). Thus, the samples were deemed suitable for data collection for high-resolution structure determination and movie frame micrographs were collected. 3D image reconstruction of 168,565 GBoV1 WT and 218,746 GBoV1 VP3-only capsids resulted in structures with an estimated resolution of 2.76 Å for both types of capsids based on an FSC threshold of 0.143 (Figure 1b–d, Table 1). 

The GBoV1 capsid structures were identical, despite being assembled from different VP compositions (Figure 1a). They share the conserved features of the *Parvoviridae* subfamily, with a channel at the 5-fold symmetry axis, protrusions around the 3-fold axis, the 2/5-fold wall, located between the depressions at the 2- and 5-fold axes and depressions at the 2-fold as well as around the 5-fold axis (Figure 1b,c) [16]. The basket-like structure beneath the 5-fold channel previously reported within the capsids of HBoV1-HBoV4 and BPV [15,23,25,26], was less pronounced in GBoV1 suggesting less order at the N-terminus of the VP (Figure 1c). This basket contains residues located at the N-terminus of VP3 and is part of a glycine-rich region hypothesized, for parvoviruses, to act as a hinge for the externalization of the VP1u to utilize its PLA2 activity. The location of this density below the 5-fold axes is consistent with the suggested use of this channel for the VP1u externalization [19,20]. Interestingly, this region of GBoV1, residues 1–32 (VP3 numbering), is similar to the analogous regions of HBoV2-4 (aa1-32) with a sequence identity ranging from 69–79% but shares only a 50% sequence identity with HBoV1 despite the higher sequence identity across the entire VP3 for all five viruses (Table 2). 

**Figure 2:**
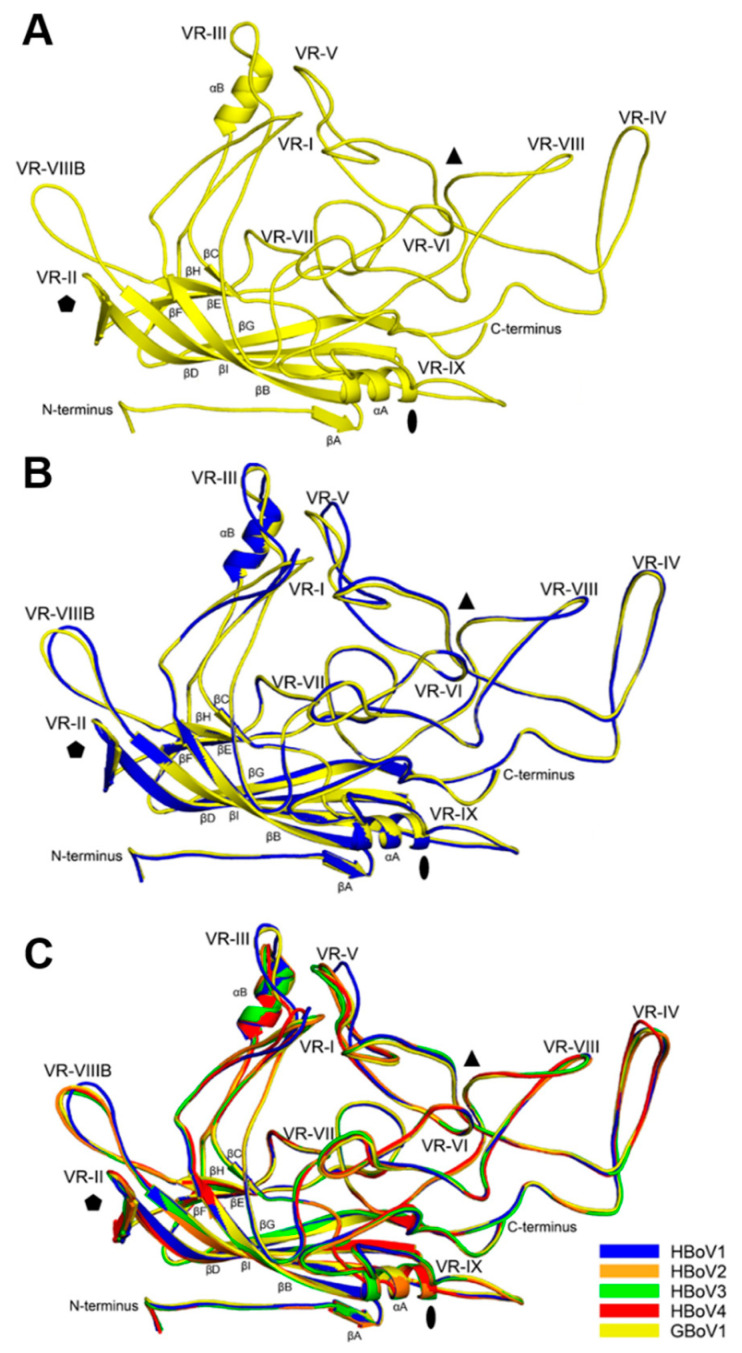
Structural comparison of GBoV1 to the HBoVs. (**A**) The VP3 monomer structure of GBoV1 shown as a ribbon diagram, with the secondary structure elements, N- and C-terminus and VRs labeled. The approximate positions of the icosahedral 2-, 3- and 5-fold axes are indicated as filled oval, triangle and pentagon, respectively. (**B**) VP3 monomer structures of HBoV1 (blue) and GBoV1 (yellow) superposed. The labels are as in panel (**A**). (**C**) VP3 monomer structures of HBoV1 (blue), HBoV2 (orange), HBoV3 (green), HBoV4 (red) and GBoV1 (yellow) superposed. The labels are as in panel (**A**). The color for each model is as given beside panel (**C**). Images were superposed in the Coot program [42] and visualized in the PyMol program [46].

In both the GBoV1 structures, excluding residues 1–32, residue 33 to the last C-terminal residue, aa542 (VP3 numbering), were structurally ordered and models could be built into the cryo-reconstructed density maps (e.g., in Figure 1e). The densities of the amino acid side chains were well-defined for the β-strands and most of the surface loops. Some acidic residue side chain densities were less defined. This observation is caused by a high sensitivity of these residue types to radiation damage as has been reported in other high-resolution cryo-constructed maps [45]. The GBoV1 VP3 structure conserved the parvovirus features, including the eight-stranded β-barrel motif, α-helix A and β-strand A (Figure 2a). The structure also featured α-helix B, a region unique to the bocaparvoviruses and the ten defined VRs, VR-I to VRVIIIB and VR-IX (Figure 2a). The GBoV1 WT model refinement statistics (Table 1) are consistent or better than for structures reported at this resolution by cryo-reconstruction for other bocaparvoviruses as well as other parvoviruses [16,23]. The root mean squared deviation (RMSD) between the VP models built into the GBoV1 WT and VP3 reconstructed density maps is 0.32 Å. Due to this high similarity, only the GBoV1 WT model will be used for further analysis. 

### 3.2. Structural Differences between GBoV1 and the HBoVs Are Localized to the Variable Regions 

**Figure 3:**
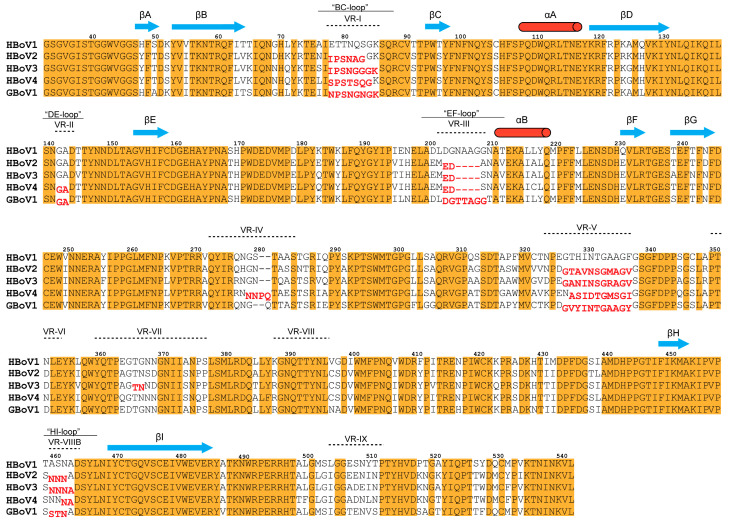
Structure-based sequence alignment of Human bocavirus 1 (HBoV1)-4 and GBoV1. The structure-based sequence alignment, starting from the first ordered residue (aa33), was generated using distance values from the Coot [42] superpose tool. Secondary structural elements, β-strands and α-helices, are indicated by blue arrows and red cylinders, respectively. Regions highlighted with orange indicate sequence identity between HBoV1-4 and GBoV1. The locations of the VRs are also indicated based on the previously defined VRs [23]. Amino acid number, based on HBoV1, is shown above the sequences. Structural variability, defined by amino acids whose Cα atoms are >2 Å apart, are offset low and highlighted in red.

The GBoV1 VP3 monomer has high primary sequence identity to the HBoVs, with 86.3% for HBoV1 and ~79% for HBoV2-4 (Table 2). The structural identity was determined by superposing the model of GBoV1 onto the previously published models of HBoV1-4 in Coot (Figure 2a–c) [23,42]. A structure-based sequence alignment was generated using these measured Cα distances, revealing that secondary structures (βI-G, βC-F core, αA and αB) are conserved and the surface loops between these regions are characterized by amino acid substitutions, insertions and deletions that result in structural differences (Figure 2 and Figure 3). 

The highest structural variabilities between the VP3s of the five viruses compared are localized to VR-I, VR-III and VR-V, with an RMSD of up to 3.3 Å, 4.5 Å and 3 Å, respectively (Figure 3, Table 3). VR-I and VR-III, along with VR-VII and VR-IX, form the 2/5-fold wall, a region of the capsid reported to be important for antigenic reactivity and receptor binding in parvoviruses [16]. VR-I has high structural variability as a result of the primary sequence differences at residues 78–85 (Figure 2b,c and Figure 3). For VR-III.

GBoV1 and HBoV1 are both structurally divergent to HBoV2-4 with a four amino acid insertion at the apex of the EF loop (Figure 3). Due to the four amino acid insertion in HBoV1 (a respiratory virus) that is not present in HBoV2-4 (gastroenteric virus) (Figure 3), VR-III was previously suggested as a region that determines tissue tropism [23]. The GBoV1 VR-III contains two amino acid substitutions relative to HBoV1 at residues 205–206 (VR-III), where NA is switched to TT (Figure 2c and Figure 3). With these substitutions, the RMSD at VR-III for GBoV1 to HBoV1 (4.5 Å) is higher than that of HBoV2-4 (3.1–3.3 Å) (Table 2). The high structural variability between GBoV1 and HBoV1-4 suggests VR-III may also play a role in host tropism. In other parvoviruses, the region analogous to VR-III serves as a determinant for tissue tropism, pathogenicity, transduction efficiency and antigenicity [24]. VR-V along with VR-IV and VR-VIII form the protrusions around the 3-fold axis. The 3-fold protrusions have been shown to be part of an antigenic footprint for HBoV1, as well as to be important for both, antigenicity and transduction efficiency in parvoviruses [16]. Interestingly, the GBoV1 VR-V is more structurally similar to the VR-V of HBoV2-4, compared to HBoV1 (Table 3). 

When comparing the GBoV1 and HBoV1 structures, RMSDs of 2.1 Å and 2.5 Å are observed for VR-II and VR-VIIIB (Figure 2b, Table 3). Differences between GBoV1 and the other viruses range from 0.6 to 2.9 Å (Table 3). VR-II is located at the apex of the DE loop, five of which form the 5-fold channel. The 5-fold channel has been proposed to be important for genome packaging and VP1u externalization [24]. VR-II is highly conserved and is part of a cross-reactive epitope between HBoV1, HBoV3 and HBoV4 [22]. The slight structural difference within the VR-II is attributed to the need for this region to be flexible to allow the proposed externalization through this 5-fold channel. VR-VIIIB or the HI loop, is located on the depressions around the 5-fold channel. While HBoV1 structurally is the most divergent compared to HBoV2-4 and GBoV1, this loop is part of a cross-reactive epitope including HBoV1, HBoV2 and HBoV4 [22]. This suggests that only a few residues within the two loops are important for the antibody recognition of the cross-reactive antibody. This region is also reported as being important for genome packaging and capsid assembly of other parvoviruses [47,48,49].

For VR-IV, VR-VI, VR-VII, VR-VIII and VR-IX, there is the least (RMSD of 1.1–0.4 Å) structural divergence of GBoV1 compared to HBoV1 (Table 3, Figure 2b). VR-IV forms the protrusions around the icosahedral 3-fold axis [23]. HBoV4 differs from the other four viruses in that it has a two amino acid insertion within this loop, conferring a different conformation to the protrusions around the 3-fold compared to the other bocaparvoviruses (Figure 2c and Figure 3). VR-VI, VR-VII and VR-VIII, all located on the side of the 3-fold protrusions, have minor to no amino acid sequence differences (Figure 3). These VRs have been shown to play a role in antigenicity and also parvovirus infectivity [16,24]. Lastly, VR-IX is located at the 2/5-fold wall. This region is structurally identical for HBoV1-4 and GBoV1, while having amino acid sequence variations and has been implicated as host tropism determinant for bocaparvoviruses [23]. As an example, BPV has a 7 amino acid deletion in VR-IX compared to the HBoVs [26]. GBoV1′s VR-IX is structurally identical to HBoV1-4 and it was shown to be capable of infecting human cell lines [33], suggesting that this region may govern primate and human cell tropisms. 

### 3.3. The GBoV1 Capsid Differs Antigenically to the HBoV1 Capsid

**Figure 4:**
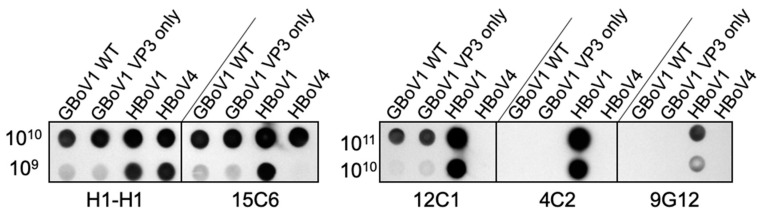
Cross-reactivity of GBoV1 capsids with HBoV1 antibodies via native dot blot. 10^11^, 10^10^ or 10^9^ viral capsids were loaded onto a nitrocellulose membrane and tested against H1-H1 (positive control for denatured virus-like particles (VLPs)) and HBoV1 antibodies 15C6, 12C1, 4C2 and 9G12 (detecting conformational epitopes). 10^11^ not shown for 15C6 and H1-H1 due to overexposure.

Antibodies 15C6, 12C1, 4G2 and 9G12 were generated, in mice, against HBoV1 capsids, using the hybridoma technology, in a previous study [22]. These antibodies were tested for reactivity against the GBoV1 capsid using native dot immunoblots (Figure 4). 15C6 and 12C1 were cross-reactive between HBoV1 and GBoV1, whereas 4C2 and 9G12 were specific for HBoV1. Previously, the 15C6 binding footprint was mapped to capsid surface features surrounding the icosahedral 5-fold axis, whereas 12C1, 4C2 and 9G12 were shown to recognize the protrusions surrounding the 3-fold axis [22]. As expected, the reactivity of these HBoV1-specific mAbs were the same for GBoV1 WT and VP3-only capsids (Figure 4). This is consistent with the observation that the epitopes of the two cross-reacting antibodies are located on the capsid surface which is formed by the VP3 common region. The antibody H1-H1, a polyclonal rabbit antibody generated against HBoV1 VP3 VLPs, served as a positive control, detecting denatured VLPs [36].

**Figure 5:**
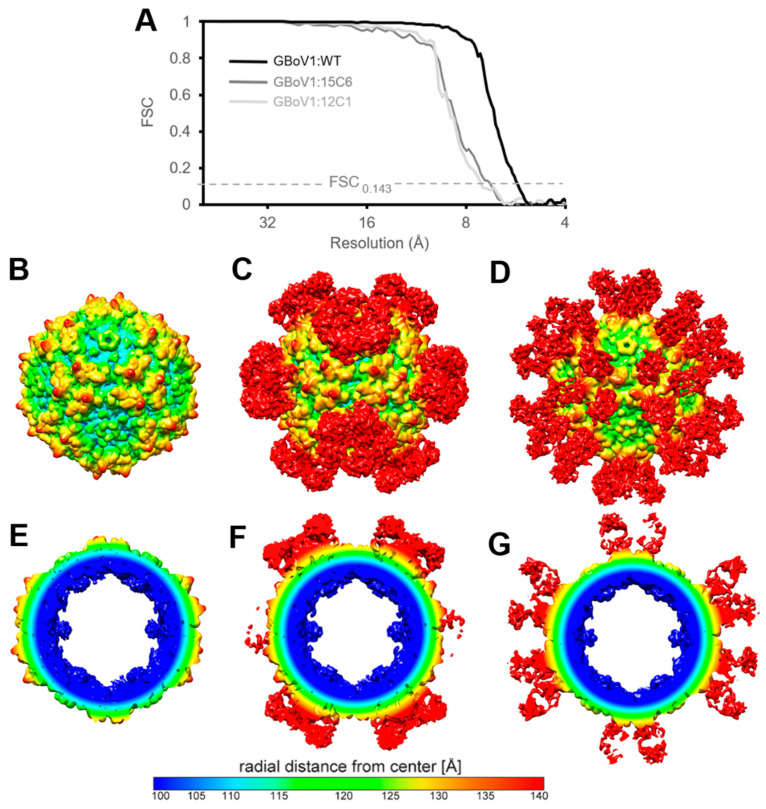
Antibody epitopes on GBoV1 capsid localized to 5- and 3-fold axes for 15C6 and 12C1. (**A**) FSC plots for the cryo-reconstruction with an estimated resolution value of 5.3Å, 6.4 Å and 6.2 Å, at an FSC threshold value of 0.143 for GBoV1, GBoV1:15C6 and GBoV1:12C1, respectively. Resolution (Å) is presented using a log_2_ scale. (**B**) Capsid density map of GBoV1 contoured at σ threshold of 1.0. (**C**) Capsid density map of GBoV1 complexed with 15C6 (GBoV1:15C6) contoured at σ threshold of 1.0. (**D**) Capsid density map of GBoV1 complexed with 15C6 contoured at σ threshold of 0.5. (**E**) Cross-sectional view of the GBoV1 complex density map. (**F**) Cross-sectional view of the GBoV1:15C6 complex density map. (**G**) Cross-sectional view of the GBoV1:12C1 capsid density map.

Cryo-EM and 3D image reconstruction were used to determine the structures of the 15C6 and 12C1 Fabs complexed with the GBoV1 WT capsid (Figure 5). A total of 1895 individual capsid complexes were used for the reconstruction of the GBoV1:15C6 and 4108 of the GBoV1:12C1 complexes, with estimated resolutions of 6.4 Å and 6.2 Å, respectively, based on a FSC threshold level of 0.143 (Figure 5a). For direct comparison, a low resolution GBoV1 WT-capsid structure was determined to 5.3 Å from 17,284 capsids (Figure 5a,b,e).

The density maps of the GBoV1:15C6 complex showed density corresponding to the bound 15C6 Fabs surrounding the 5-fold channel (Figure 5c). For the GBoV1:12C1 complex, the 12C1 Fab was bound on the protrusions surround the 3-fold axis (Figure 5d). A 0.5 σ threshold density map was used to visualize both, the complimentary-determining regions (CDR) and the constant regions of the Fab (Figure 5d). At 1σ threshold, five copies of the Fab are visible of the GBoV1:15C6 complex but only the CDR for the GBoV1:12C1 complex (not shown). The visible surface features, 3-fold protrusions and 2/5-fold wall, for the GBoV1:15C6 complex is consistent with the low-resolution GBoV1 WT structure. The 5-fold channel and depressions surrounding the channel is also consistent in the GBoV1:12C1 complex with the low-resolution GBoV1 WT structure (Figure 5b). Underneath the 5-fold channel, a basket-like density can be seen in all three structures (Figure 5e–g), consistent with improved ordering at lower resolution as observed in the low- and high-resolution structures of BPV and HBoV1-4 [15,23]. 

**Figure 6:**
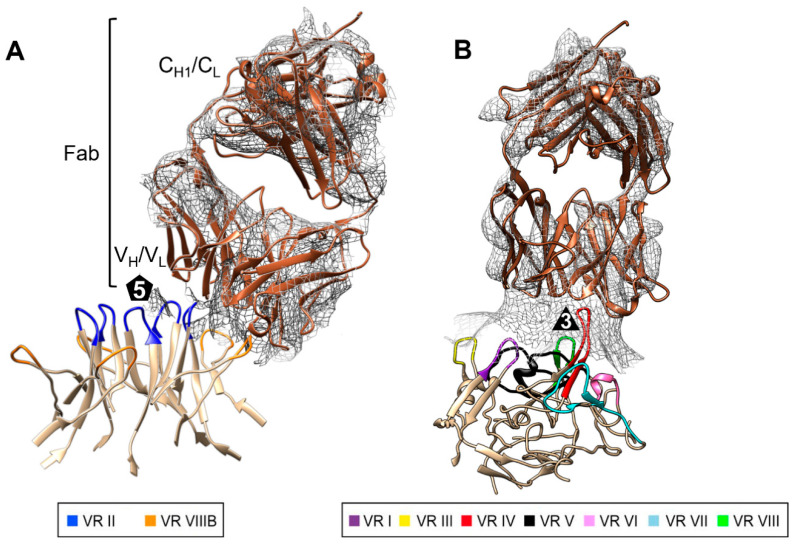
GBoV1-Fab binding interfaces. (**A**) Close-up view of the GBoV1 WT structure docked to a generic Fab (PDB ID 2FBJ) within the cryo-reconstructed density of GBoV1-15C6 (represented as a gray mesh, contoured at 0.5σ) and (**B**) GBoV1-12C1. Highlighted VRs are colored as shown in key. Generic Fab (dark brown) consists of a heavy and light chain, each with constant and variable regions. The Fab variable region interacts with the surface of the capsid. The GBoV1 capsid is also colored in tan.

**Figure 7:**
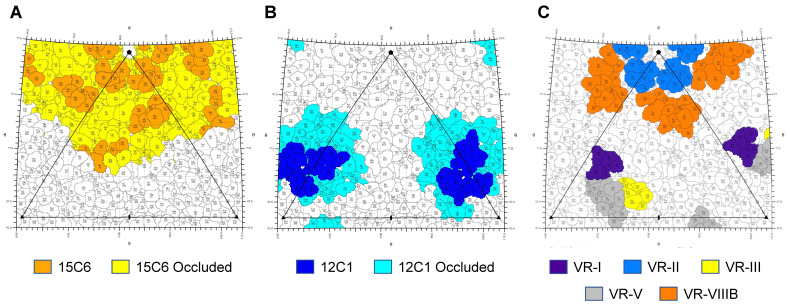
The GBoV1 15C6 and 12C1 epitopes. (**A**) Roadmap surface representation of the GBoV1 15C6 epitope. Colored in orange are the modeled contact residues between the GBoV1 capsid and the 15C6 Fab model. Colored in yellow are the residues occluded by the bound 15C6 Fab. (**B**) Roadmap surface representation of the GBoV1 12C1 epitope. Colored in blue are the modeled contact residues between the GBoV1 capsid and the 12C1 Fab model. Colored in cyan are the residues occluded by the 12C1 Fab. (**C**) Position of VR-I, VR-II, VR-III and VR-V, VR-VIIIB on the GBoV1 capsid. Amino acid residues that are exposed on the capsid surface are labeled with their 3-letter code and residue number. The 5-fold, 3-fold and 2-fold axis are indicated by a filled pentagon, triangle and ellipse, respectively. The roadmaps were generated with the RIVEM program [44].

Rigid-body docking of the refined 60-mer model of GBoV1 WT was performed for the GBoV1:15C6 and GBoV1:12C1 density maps, along with a generic Fab (PDB ID: 2FBJ), with a CC of 0.93 and 0.90, respectively (Figure 6). Steric clashes at the 5-fold axis for the individual 15C6 Fabs likely resulted in local disorder for the Fab (Figure 6). For 12C1 it appears that enough space is available at the 3-fold protrusions (Figure 5) but disorder also is observed. A 2D stereographic projection (roadmap) representation of the complex, generated based on the fitted models, identified the contact and occluded (within Fab footprint but not contacting capsid) residues (Figure 7) [44]. The 15C6 epitope lines the 5-fold channel, encompassing the residues that form VR-II (residues 142-GAD-144) and VR-VIIIB (residues 460-STNA-463), which are the DE and HI loops, respectively (Figure 7a). These residues are conserved between GBoV1 and HBoV1, with the exception of residue S460, which is A460 in HBoV1. The high conservation within this sequence region as well as high structural similarity explains the cross-reactivity between the GBoV1 and HBoV1 capsid for the 15C6 antibody. This antibody also cross-reacts with HBoV2 and HBoV4 [22]. The 12C1 epitope sits on the protrusions around the 3-fold axis and contains contact residues from VR-I (80-SNGN-83), VR-IV (276-IRQNGQTTA-284) and VR-VIII (390-NQTT-393) (Figure 7b). Compared to HBoV1, the GBoV1 VR-IV and VR-VIII are structurally identical despite having amino acid differences (Figure 3). This structural identity likely dictates the cross-reactivity of 12C1 for the GBoV1 capsid. Interestingly, antibodies 4G2 and 9G12, also recognizing the 3-fold protrusions, were not cross-reactive despite the 94.3% structural identity that is shared between the GBoV1 and HBoV1 VP3 monomers [22]. VR-I, VR-III and VR-V are the most structurally divergent loops between HBoV1 and GBoV1 and contain residues outside the 15C6 and 12C1 epitopes (Figure 7c). These residues outside these epitopes, particularly at the 3-fold protrusion, are potentially responsible for this difference in antigenic reactivity with respect to 4G2 and 9G12. 

### 3.4. HBoV1 and GBoV1 Share Similar Rates of Seropositivity

**Figure 8:**
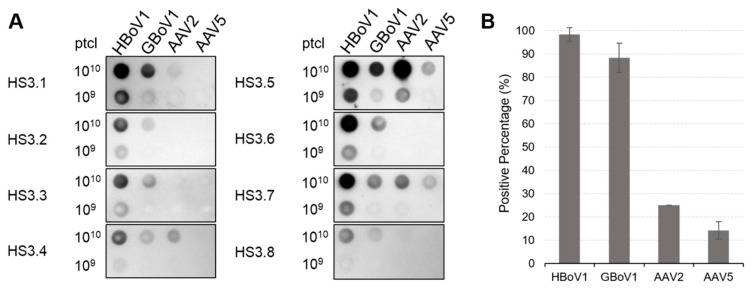
Dot immunoblot analysis of HBoV1 and GBoV1 against human sera. (**A**) Representative native dot immunoblots of HBoV1 and GBoV1 against human sera with 10^10^ or 10^9^ loaded capsid particles. AAV2 and AAV5 are used as controls. Samples tested are as labeled. (**B**) Bar graph representation of the percentage of positive signal based visual inspection of the 40 dot immunoblots reactivities. *n* = 3.

Forty human serum samples from adult donors were screened by native dot immunoblot against HBoV1, GBoV1, AAV2 and AAV5 capsids. Approximately 98.3% of the samples reacted to HBoV1 capsids, 88.3% against GBoV1, 25% against AAV2 and 14.2% against AAV5, respectively (Figure 8). This suggests that HBoV infections are prevalent in North America, the source of the analyzed human sera. This seroprevalence is comparable to results from a previous study analyzing sera from adults in Finland (95%) and Pakistan (99%) [50]. Nevertheless, seroprevalences are obtained without consideration of HBoV2-4 cross-reacting IgGs, so the true rates of HBoV1-specific and GBoV1-specific seropositivity will be skewed by potential HBoV2-4 IgGs. The high GBoV1 response is thus likely caused by anti-HBoV antibodies. In addition, generally weaker signal intensities were observed for GBoV1 compared to HBoV1 across the forty samples (Figure 8). The minor difference of 10% in seropositivity indicates variation in antigenic reactivity. Interestingly, AAV2 has been reported to be have a 72% seroprevalence (59% neutralizing) in French adults, a significant difference compared to the data here, suggesting location may be a large variable in these epidemiological studies [51]. As an example, another study reported 25–30% neutralizing antibodies against AAV2 when 100 human serum samples from North American adults were tested [52]. It is important to note that native dot immunoblots do not report the neutralization potential of the antibodies from the forty samples but rather the antibodies capable of recognizing the capsid surface. Further study of neutralizing factors and cross-reactivity within the human sera will be needed to determine the effect of such samples on the transduction efficiency in human cells and tissue. 

## 4. Conclusions

This study reports the first high-resolution structure of the GBoV1 WT capsid, resolved to 2.76 Å resolution. Compared to other members of the genus, the GBoV1 capsid shares similar surface features, such as the channel at the 5-fold symmetry axis, protrusions around the 3-fold axis, the 2/5-fold wall, located between the depressions at the 2- and 5-fold and depressions at the 2-fold as well as around the 5-fold axes. In addition to the high-resolution WT structure, the structures of two capsid-antibody complex structures are reported, highlighting antigenic epitopes on the GBoV1 capsid surface. Both, GBoV1 and HBoV1 share a high sequence and structural identity, with major structural differences localized to VR-I, VR-III and VR-V. VR-I and VR-III are both part of the 2/5-fold wall of the capsid and VR-V is located on the protrusions around the icosahedral 3-fold. These VRs contain residues that are within the epitopes of HBoV1 cross-reactive monoclonal antibodies 12C1, 4C2 and 9G12. All three antibodies share the same antibody epitope, as previously reported [27]. Interestingly, native dot immunoblots show that the GBoV1 capsid is capable of escaping 4C2 and 9G12 but not 12C1, suggesting that minor structural differences at these VRs are responsible for the GBoV1 capsid’s ability to escape antibodies 4C2 and 9G12. Overall, the GBoV1 and HBoV1 capsid are antigenically similar at the icosahedral 5-fold axis, a region that is most conserved amongst parvoviruses, yet differ at the 3-fold axis. The reported capsid structures and epitopes can guide strategies for vector engineering and aid to develop the GBoV1 capsid structure as a viral vector. In addition, the HBoV1 and GBoV1 capsid seropositivity rates against human sera points to high cross-reactivity between the two viruses. Potential binding sites are likely the 5-fold region that is highly conserved. This, however, remains to be determined. Interestingly, the HBoV1 and GBoV1 seropositivity rates with human sera were significantly higher than those for AAV2 and AAV5 in the North American adult samples tested. This observation further emphasizes the need to understand the antigenic reactivity of the bocaparvoviruses if they are to be developed as vectors for clinical gene delivery. 

## Figures and Tables

**Table 1:** Summary of data collection, processing and refinement statistics.

Parameter	GBoV1
Total no. of micrographs	1411
Defocus range (μm)	1.08–3.19
Electron dose (e^−^/Å^2^)	60
No. of frames/micrograph	50
Pixel size (Å/pixel)	1.08
No. of capsids used for final map	168,565
Resolution of final map (Å)	2.76
PHENIX model refinement statistics	
Residue range	33–542
Map CC	0.877
RMSD (Å)	
Bonds	0.01
Angles	0.89
All-atom clash score	10.61
Ramachandran plot (%)	
Favored	98.4
Allowed	1.6
Outliers	0.0
Rotamer outliers	0.0
No. of Cβ deviations	0

**Table 2:** HBoV1-4 and GBoV1 primary VP3 sequence identity (bottom left) and structural identity (top right).

	HBoV1	HBoV2	HBoV3	HBoV4	GBoV1
HBoV1		94.7	93.9	93.8	94.1
HBoV2	77.5		98.8	97.4	98.2
HBoV3	77.2	89.2		97.3	96.5
HBoV4	77.2	88.7	90.2		95.3
GBoV1	86.3	79.7	79.9	79.4	

**Table 3:** Local root mean squared deviations (RMSDs) in angstroms (Å) for aligned HBoV1-4 and GBoV1 VRs. Higher values are shaded darker.

	VR-I	VR-III	VR-V	VR-II	VR-VIIIB	VR-IV	VR-VI	VR-VII	VR-VIII	VR-IX
HBoV1 vs. HBoV2	3.1	3.1	2.9	1.9	1.8	1.1	0.7	1.1	0.7	1.0
HBoV1 vs. HBoV3	2.9	3.2	3.6	1.3	2.9	0.9	0.4	1.3	0.6	1.3
HBoV1 vs. HBoV4	2.8	3.3	3.0	2.6	2.5	2.1	1.1	1.2	1.2	1.1
HBoV1 vs. GBoV1	3.3	4.5	3.0	2.1	2.5	0.9	0.4	1.1	0.7	1.1
HBoV2 vs. HBoV3	1.8	1.0	1.5	1.4	1.6	0.8	0.8	0.8	0.9	0.6
HBoV2 vs. HBoV4	2.9	1.2	1.5	1.2	1.6	1.6	0.5	0.8	1.1	0.7
HBoV2 vs. GBoV1	0.8	3.4	1.2	0.6	0.6	0.9	0.6	0.7	0.7	0.2
HBoV3 vs. HBoV4	2.9	0.9	1.5	1.4	1.3	2.0	0.7	0.9	1.3	0.6
HBoV3 vs. GBoV1	2.0	3.3	1.8	1.8	1.7	0.9	0.5	0.9	0.9	0.8
HBoV4 vs. GBoV1	3.2	3.8	1.2	0.9	1.5	1.8	0.6	0.8	0.9	0.3
